# Standardized Full-Mouth Rehabilitation Using an Innovative Digital Workflow for Patients with Severe Dental Erosion—A Retrospective Case Series on Functional, Aesthetic, and Patient-Reported Outcomes

**DOI:** 10.3390/dj14070407

**Published:** 2026-07-05

**Authors:** Polina Kotlarenko, Tom Vaskovich, Astrid Skolka, Andreas Moritz, Alexandra Thajer

**Affiliations:** 1 Division of Conservative Dentistry and Periodontology, University Clinic of Dentistry, Medical University of Vienna, Sensengasse 2a, 1090 Vienna, Austria; polina.kotlarenko@meduniwien.ac.at (P.K.); andreas.moritz@meduniwien.ac.at (A.M.); 2 Dental Technical Laboratory, University Clinic of Dentistry, Medical University of Vienna, Sensengasse 2a, 1090 Vienna, Austria; tom.vaskovich@meduniwien.ac.at; 3 Division of Prosthodontics, University Clinic of Dentistry, Medical University of Vienna, Sensengasse 2a, 1090 Vienna, Austria; astrid.skolka@meduniwien.ac.at; 4 Division of Pediatric Pulmonology, Allergology and Endocrinology, Department of Pediatrics and Adolescent Medicine, Comprehensive Center for Pediatrics, Medical University of Vienna, Währinger Gürtel 18–20, 1090 Vienna, Austria

**Keywords:** bulimia nervosa, dental erosion, digital dentistry, full-mouth rehabilitation, vertical dimension of occlusion, CAD/CAM

## Abstract

**Background/Objectives**: The aim of this study was to show a standardized four-step technique that can offer individually personalized full-mouth therapy for each complex dental patient with erosive tooth wear resulting from bulimia nervosa, focusing on the individualized vertical dimension of occlusion (VDO), functional and aesthetic stability, and patient-reported outcomes, including dental symptoms, nutrition, self-perception, and quality of life. **Methods**: The following steps are proposed for structured full-mouth rehabilitation. Step 1: Intraoral diagnosis via a single computer-aided impression. Step 2: Determination of a new adequate vertical dimension of occlusion and soft tissue prediction. Step 3: Removable sample dentures—prototypes. Step 4: Non-prep/minimal-prep crowns as the long-term provisional/definitive treatment. **Results**: Nine adults (11% male) with dental erosion caused by bulimia nervosa (78%), gastro-esophageal reflux (11%), and soft drinks (11%) were part of this cohort. The novel digital workflow enabled restoration of an individualized vertical dimension of occlusion, stable occlusion, appropriate centric and eccentric contacts, biomimetic dental anatomy, harmonious tooth proportions, and optimized red–white aesthetics. Dental problems (hypersensitivity, dental pain), nutritional behavior, body perception, and quality of life improved after the full-mouth rehabilitation. **Conclusions**: The presented digital workflow offers a promising approach for full-mouth rehabilitation in patients with severe dental erosion, particularly associated with bulimia nervosa, enabling structured restoration planning and stepwise evaluation of the vertical dimension of occlusion and functional adaptation. Prospective studies with larger cohorts are needed to confirm long-term clinical outcomes and patient-reported benefits.

## 1. Introduction

Dental erosion is defined as the irreversible loss of dental hard tissues, specifically enamel and dentin, caused by chemical dissolution from intrinsic or extrinsic acids, independent of bacterial activity [1,2,3,4,5]. Intrinsic etiologies include gastric acid exposure from vomiting, as seen in bulimia nervosa, or gastroesophageal reflux, a recognized contributor to erosive tooth wear [6,7,8,9]. Extrinsic etiologies involve chronic consumption of acidic foods and beverages [2,6,10].

Severe erosion frequently affects multiple teeth, leading to diminished crown height, hypersensitivity, pulp exposure, occlusal alterations, and compromised dental and facial aesthetics [1,2,3,6,7,9,11,12]. Progression of the erosive lesions might lead to permanent damage to function and aesthetics. These changes in dental and facial aesthetics and oral function have a major impact on patients with bulimia nervosa.

Bulimia nervosa (BN) is an eating disorder marked by recurrent binge eating followed by purging, such as self-induced vomiting, to prevent weight gain. It primarily affects female adolescents and often persists for years [13,14,15,16]. Body perception, especially perception of the oral region, is impaired in patients with bulimia nervosa; it is a disorder that takes place in the oral region, as this is the region where the excessive dietary intake and self-induced vomiting are carried out. Early clinical signs include embrittled upper anterior teeth with marked reduction in tooth length, accompanied by functional deficits in mastication, phonetics, muscular tension, and temporomandibular joint (TMJ) comfort. Aesthetic concerns arise from reductions in the vertical dimension of occlusion (VDO), which reflects the vertical height of the lower third of the face when the upper and lower dental arches are in maximum intercuspation [17,18]. Reductions in VDO due to tooth wear or loss of posterior support can alter facial expression, affect occlusal harmony, compromise phonetics, and decrease neuromuscular comfort [17]. These changes exacerbate the negative impact of dental erosion on self-esteem and quality of life, particularly in BN patients.

At this point, it might be necessary to replace or restore every tooth in the mouth, defined as full-mouth rehabilitation (FMR) [19,20,21,22].

Full-mouth rehabilitation is a complex restorative therapy intended to change function and aesthetics through a change in the bite situation and/or occlusion surface and guidance area and/or vertical dimension of occlusion and/or sagittal or transversal dimension. This change in function and aesthetics represents a massive intervention for the affected patients. Patients with a body scheme disorder, in particular, are desperate to address their current oral situation of brittle, reduced front teeth, which have an impact on their smile. These patients seek a smile makeover but, at the same time, have severe difficulties with accepting any change because of their disturbed body perception. Hence, conservative full-mouth rehabilitation is not feasible in patients suffering from this eating disorder.

Moreover, patients presenting with severe dental erosion associated with bulimia nervosa represent a highly specific and underreported clinical population. Due to the stigma associated with eating disorders, many patients are reluctant to disclose their condition, which limits the availability of well-documented clinical cohorts. Furthermore, full-mouth rehabilitation cases involving a controlled increase in the vertical dimension of occlusion are complex and relatively rare in clinical research [23,24,25]. To our knowledge, only limited clinical data based on single case reports are available describing digitally guided full-mouth rehabilitation protocols in patients with severe dental erosion associated with bulimia nervosa [26,27].

Therefore, a novel method was developed specifically for patients with bulimia nervosa, supported by planned and systematically evaluated dental, facial, and smile-related outcomes, which facilitates acceptance of future dental restorative therapy during a testing phase. Any massive intervention in dental therapy also contains a certain risk of failure, which would represent, in FMR, the ultimate maximum credible accident (MCA). It is already a failure if the outcome shows any discrepancy between dentist and patient expectations, leading to negative consequences and time and financial loss on both sides. To increase the likelihood of success in complex restorative therapy, maximum predictability of aesthetics and function should be ensured, and patients should be involved in the decision-making process, including non-medical cosmetic choices such as future tooth color, to facilitate acceptance of the outcome in individuals with body image disorders. In consequence of erosive tooth loss, the lower face height is reduced, and full-mouth rehabilitation with additive tooth length must be implemented in a new adequate vertical dimension of occlusion. The future VDO of the patient can be determined by means of skeletal analysis through cephalometry, and a virtual preview of changes due to modifications to the lower facial height can be obtained. In addition, to evaluate the factors associated with facially driven soft tissues of the face, like lip closure and the lateral profile, a novel standardized soft tissue prediction using splints in different thicknesses has been developed [28]. This soft tissue prediction allows the dentist to analyze the soft tissue of the frontal and lateral views and to co-determine with the patient their new VDO from feedback on their impressions, optical and sensoric, with the different splints. This guarantees the acceptance of the novel VDO in patients with body scheme disorders.

To address these challenges, a novel protocol was developed to provide facially driven prediction as well as virtual and clinical methods to improve the reliability of treatment planning and outcome assessment of full-mouth rehabilitation in patients with dental erosion, particularly in individuals with bulimia nervosa. This structured full-mouth rehabilitation causes massive oral changes. It is therefore essential to examine the changes in these patients, especially in psychologically imbalanced patients with erosion caused by bulimia nervosa, in relation to their diet, body perception (especially that of facial and oral changes), and quality of life.

The aim of this study was to present a standardized four-step protocol for providing individually tailored full-mouth rehabilitation in patients with erosive tooth wear, particularly associated with bulimia nervosa, with a focus on the individualized vertical dimension of occlusion, functional and aesthetic stability, and patient-reported outcomes, including dental symptoms, nutritional behavior, self-perception, and quality of life.

## 2. Materials and Methods

### 2.1. Study Design

This study retrospectively reviewed the clinical records of adults who were treated using the digital workflow for structured full-mouth rehabilitation at the University Clinic of Dentistry in Vienna, Austria. The aim of the study was to describe the clinical workflow of the digitally guided rehabilitation protocol and explore both clinical and patient-reported outcomes following treatment.

### 2.2. Study Patients

The inclusion criteria for the study comprised patients over 18 years of age, diagnosed with dental erosion caused by bulimia nervosa, gastrointestinal disorders, or nutritional failures, who underwent treatment using the digital workflow for structured full-mouth rehabilitation at the dental outpatient clinic for bulimia nervosa and dental erosion, University Clinic of Dentistry, Medical University of Vienna, between 7 January 2013 and 23 December 2020. The exclusion criteria comprised children and adolescents and patients presenting with dental erosions resulting from amelogenesis, dental aplasia, bruxism, or a loss of dental hard tissue of unknown origin, as well as patients presenting with temporomandibular joint (TMJ) disorders.

### 2.3. Outcomes

The primary outcome was the change in vertical dimension of occlusion (VDO), defined as the difference in millimeters (mm) between baseline and definitive full-mouth rehabilitation. VDO was quantified using predefined increments of +5 mm, +7 mm, and +10 mm, providing a standardized and reproducible measure to assess the effectiveness of the intervention across study populations. Secondary outcomes included patient-reported measures, specifically dental symptoms (pain and hypersensitivity), self-reported nutritional behavior, body perception related to facial and dental appearance, and overall quality of life. All outcome parameters were assessed at baseline, and patients were reevaluated following completion of the FMR.

### 2.4. Novel Concept for Structured Full-Mouth Rehabilitation Using a Digital Workflow in Patients with Dental Erosion

The Austrian Dental Bulimia Nervosa Group designed an innovative concept of predictable full-mouth rehabilitation. The four-step digital workflow supports a structured and reproducible clinical approach by enabling objective evaluation at multiple stages. Accurate digital replication of the intraoral situation, individualized VDO adjustment, and a non-invasive test phase with removable prototypes allow both functional and aesthetic verification while incorporating patient feedback. Final restorations fabricated with non-prep and minimal-prep crowns derived from the validated prototype achieve high precision in a single session, reducing technical variability and supporting consistent clinical execution. By systematically verifying each stage prior to finalization, the workflow minimizes uncertainty, aligns patient and clinician expectations, and maximizes the reliability of functional and aesthetic evaluation, even in complex full-mouth rehabilitation cases. The workflow allows stepwise verification and clinical assessment at multiple stages, as follows:

Step 1 (CAI): Ensures accurate digital replication of the intraoral situation.
Step 2 (VDO determination): Combines cephalometric measurements and soft tissue analysis for individualized and clinically assessed vertical dimension adjustment.
Step 3 (Removable sample dentures): Provides a non-invasive test phase, allowing functional and aesthetic verification before final restorations. Patient feedback during this phase is a key predictor of treatment acceptance.
Step 4 (Non-prep/minimal-prep crowns): Uses the previously validated prototype design for precise, biologically conservative restorations in one session, minimizing variability.

A case report of this innovative concept has already been published [26]. However, the following text describes the full documentation of the novel four-step technique, which was supported by a virtual articulator (ceramill Artex^®^CR, Amann Girrbach, Pforzheim, Germany).

**Figure 1:**
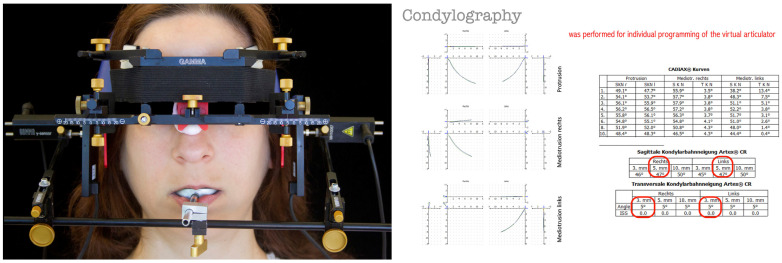
Electronic condylography and the data for individual programming.

For every patient, electronic condylography (CADIAX^®^4, Gamma dental, Klosterneuburg, Austria) was conducted, and the values gained were used to individually program the virtual articulator (ceramill Artex^®^CR, Amann Girrbach, Pforzheim, Germany) (Figure 1).

The electronic condylography (CADIAX^®^4, Gamma dental, Klosterneuburg, Austria) helped with determination of the precise hinge axis point and registration of the open joint tracks.

A complete intraoral status assessment was performed to evaluate the mobility and sensitivity of teeth. The temporomandibular joint status and periodontal status were determined, and X-rays were taken. Computer tomography was conducted if necessary.

- Step 1: Intraoral diagnosis from a single computer-aided impression.

**Figure 2:**
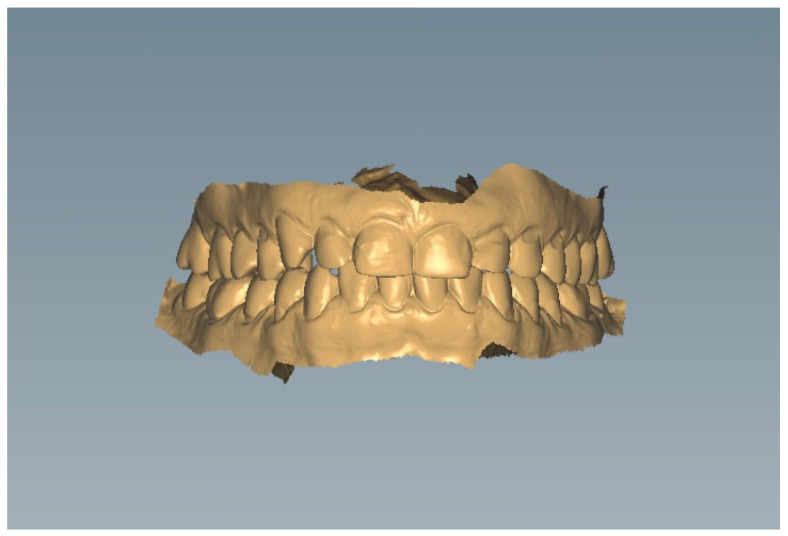
A CAI of the initial situation transferred to Ceramill^®^ Mind software.

The first step is to obtain a computer-aided impression (CAI) and a computer-aided bite registration. The CAI is used for digital transfer in the planning software (ceramill^®^ mind, Amann Girrbach, Pforzheim, Germany) (Figure 2).

With this process, an intraoral diagnosis followed by treatment planning is feasible. This computer-aided impression is used with the support of computer-aided design (CAD)/computer-aided manufacturing (CAM) techniques to obtain an impression of the future restoration of function and aesthetics. The novelty of this method is the patient-oriented procedure, in that only one CAI is needed to design and manufactur splints in different vertical dimensions and facially driven removable sample dentures (prototypes/mock-ups). For the patient, a single-impression technique enables an immense reduction in the number of overstrained appointments, giving the patient a preview of the future restoration without any further pre-therapy dental intervention. The patient can see, with the support of step 3 (removable sample dentures), the presumed changes in the final outcome, which is a factor in the predictability of function and aesthetics.

- Step 2: Determination of a new adequate vertical dimension of occlusion.

In cases of severe tooth wear, most teeth are affected by erosion and predominantly hard dental tissue has been lost, with the consequence of a decreased lower facial height. The aim is facially driven oral reconstruction of the vertical dimension of occlusion. Moreover, there is a need for space for the reconstructions with crowns on non-prepped teeth. Therefore, it is essential to define a new adequate vertical dimension of occlusion individualized for each patient with severe erosion. The determination of a new adequate vertical dimension of occlusion is supported by a cephalometric analysis (Cadias^®^, Gamma Dental Software, Klosterneuburg, Austria) as well as a “soft tissue prediction” technique [28].

**Figure 3:**
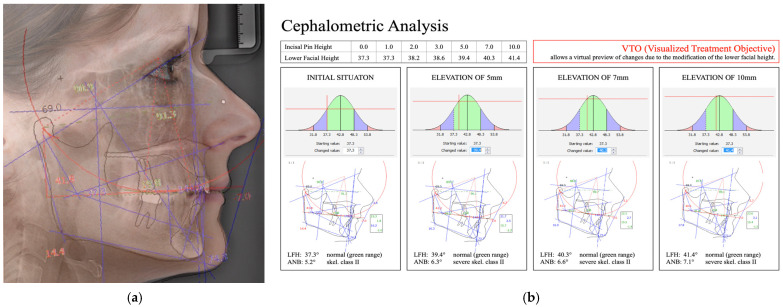
(**a**) Cephalometric analysis. (**b**) Virtual previews of changes due to modification of the lower facial height. Green indicates values within the normal range, blue indicates values outside the normal range, and red indicates values that are extremely outside the normal range.

The cephalometric analysis provides, on the one hand, the numeric values of the present situation, but allows, on the other, a virtual preview of changes due to modification of the lower facial height (Figure 3) [26,29,30].

The cephalometric analysis of the initial situation involves a fusion of lateral profile photography and lateral cephalometric X-ray, with illustrated points and structures (Cadias^®^, Gamma Dental Software, Klosterneuburg, Austria).

**Figure 4:**
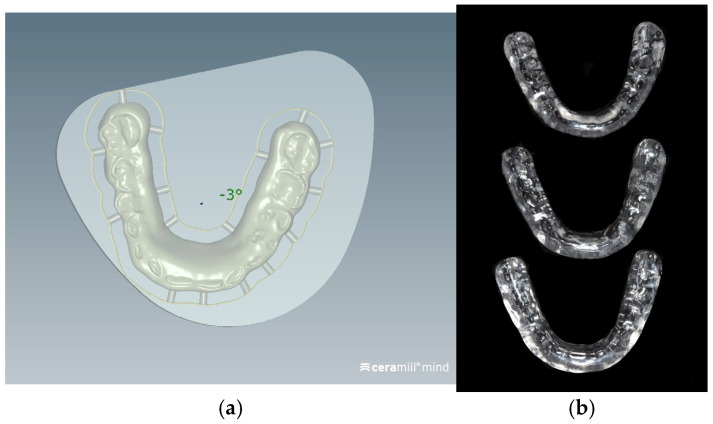
(**a**) CAD/CAM splints; (**b**) different thicknesses.

The “soft tissue prediction” technique is based on facial analysis, increasing the vertical dimension through the addition of intraorally positioned CAD/CAM splints (ceramill^®^ splintec, Amann Girrbach, Mäder, Austria) in different thicknesses (+5 mm, +7 mm, +10 mm incisal pin) (Figure 4).

**Figure 5:**
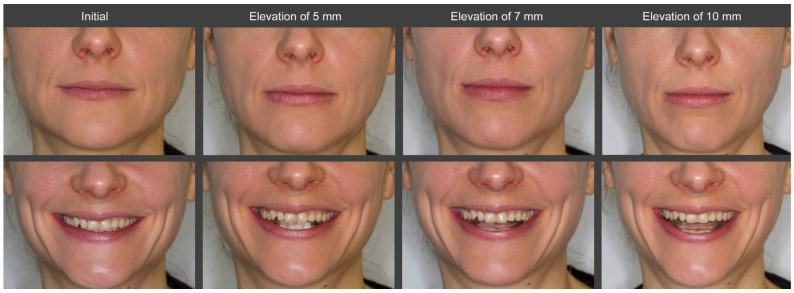
Soft tissue prediction. The patient’s initial situation and elevation of the vertical dimension of occlusion by +5 mm, +7 mm, and +10 mm are illustrated from left to right. The top row of images presents the patient with a closed mouth. With the proportional increase in the VDO, increased tension of the mentalis muscle and expanded lip vermillion exposure can be observed. This serves to control if efficient lip closure is still feasible with a relaxed muscle tone of the perioral muscles. The bottom row of pictures shows the patient smiling. This helps to illustrate how the increase in the VDO affects direct proportional changes in the oral display, perioral muscle tension, aesthetic facial appearance, and lower face height.

The facial analysis is performed for the front view and the lateral profile concerning aesthetic (expanded exposure of the vermillion of the lips, aesthetic facial appearance, and proportions of the face) and functional (perioral muscle tension and if lip closure is still possible) parameters (Figure 5).

In every patient, the rise in the vertical dimension of occlusion into the appropriate range could first be seen through the individual cephalometric analysis and then needs to be confirmed and adjusted via soft tissue control, defined by the expression “soft tissue prediction” [28]. The future vertical dimension of occlusion is defined in accordance with the subjective perception of the patient with regard to comfort and with the analysis of the above mentioned functional and aesthetic parameters by the dentist.

- Step 3: Removable sample dentures—prototypes.

**Figure 6:**
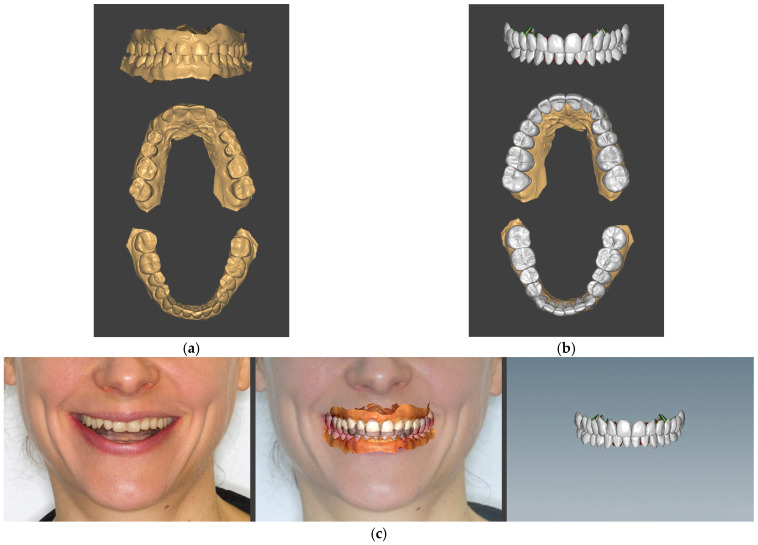
Facially driven tooth set-up. (**a**) initial situation (**b**) virtual tooth set up supported by CAD (**c**) from left to right: left: patient’s initial status with splint in new vertical dimension of occlusion. in the middle: overlapping the initial status with the CAD of the (**b**); and right: virtual tooth set up supported by CAD.

The CAI from step 1 is used for the computed-aided design and manufacture of facially driven removable sample dentures (prototypes/mock-ups). The CAI of the initial situation and the digital teeth are set-up for a new VDO (Figure 6a,b). Overlapping the 2D picture with a splint for an elevated VDO with the intraoral scan for facially driven CAD of the tooth set-up provides a preview of the future oral display (Figure 6c).

**Figure 7:**
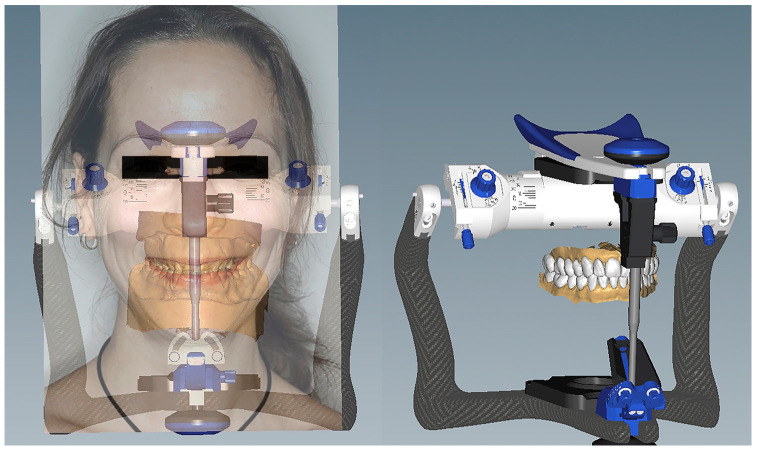
Facially guided digital treatment planning.

Facially guided digital treatment planning matches the facial analysis with the intraoral scan and the virtual articulator (Figure 7, left). The digital tooth set-up is constructed in the virtual articulator (Ceramill^®^ Mind, Amann Girrbach, Pforzheim, Germany) (Figure 7, right).

**Figure 8:**
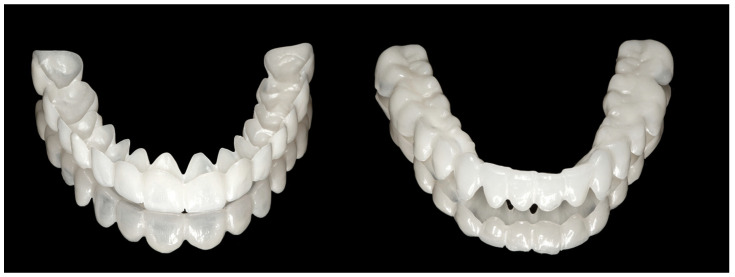
CAD/CAM milled removable sample dentures.

The CAD/CAM overdentures are milled out of polymethyl methacrylate (PMMA) by using a five-axis milling machine (Ceramill^®^ Motion, Amann Girrbach, Pforzheim, Germany) (Figure 8) [26].

Beginning with the final outcome in mind, removable sample dentures (prototypes) give a non-invasive facially driven preview of functional and aesthetic changes designed and produced on the CAI of the present situation and support a non-invasive introduction to the new vertical dimension of occlusion (VDO). A settling phase is recommended in which the patient wears the sample dentures controlling function and aesthetics to encourage acceptance of the facial changes.

Following a non-invasive testing phase averaging three months, the patient was given sufficient time to adapt to the intra- and extraoral changes introduced by the trial dentures and to evaluate social feedback related to these novel oral and facial modifications. The trial prototypes could be worn for up to 24 h per day, with a minimum daily wearing time of seven hours recommended to facilitate neuromuscular and temporomandibular adaptation. One main inclusion criterion for all patients in the cohort group to undergo the novel four-step technique was having no TMJ problems.

- Step 4: Non-prep/minimal-prep crowns as long-term provisional.

**Figure 9:**
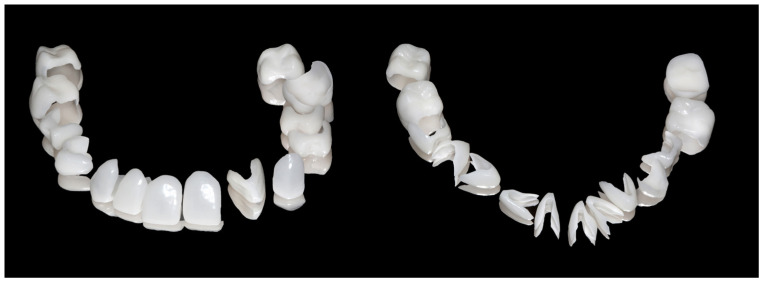
CAD/CAM milled monolithic single crowns.

Biological structures are maintained by applying a non-prep/minimal-prep method; i.e., bonding CAD/CAM single crowns onto unprepared or minimally prepared teeth (Figure 9).

**Figure 10:**
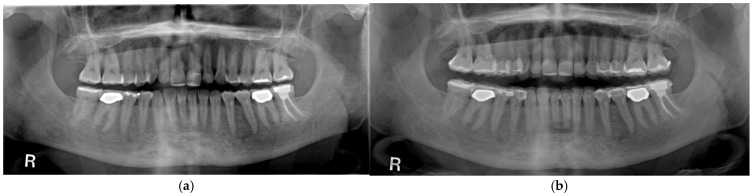
(**a**) Initial radiograph. (**b**) Final radiograph with CAD/CAM single crowns bonded onto unprepared teeth and pre-existing restorations.

After the patients’ acceptance of the prototypes, the prototype data (CAD) are utilized for CAM of the final rehabilitation. The same design is reused but in the form of single crowns or a similar-looking fixed or removable rehabilitation. These crowns are bonded as the new full-mouth restoration in only one appointment. The CAD/CAM single crowns are milled out of the hybrid material ENAMIC^®^ (VITA Zahnfabrik, Bad Säckingen, Germany) and bonded with 3M™ ESPE™ RelyX™ (3M, Kamen, Germany) or Multilink Automix (Ivoclar Vivadent, Schaan, Liechtenstein). ENAMIC^®^ (VITA Zahnfabrik, Bad Säckingen, Germany) is a hybrid dental ceramic with a dual network structure (ceramic and acrylate polymer). The adhesive procedure can be performed on natural tooth structures and on pre-existing restorations if they are free from carious lesions (Figure 10).

### 2.5. Treatment Plan for Patients with Dental Erosion

Full-mouth therapy with restoration of missing tooth structures is performed to correct function and aesthetics, supported by a digital workflow and a four-step technique. The following table shows the transition from the demands of a complex case to the solution supported by the four steps (Table 1).

### 2.6. Nutritional Analysis

The nutritional behavior of the patients was evaluated at two time points: at baseline and at the last full-mouth rehabilitation visit. The nutritional evaluation was based on a 24 h recall. Interviews were conducted by a trained nutritional expert to examine the patients’ nutritional behavior before and after the dental FMR. Throughout the interviews, patients were asked about their food and drink intake, as well as their physical activity. Furthermore, bulimia nervosa patients were asked about their frequencies of binge eating and self-induced vomiting episodes. The recorded information about daily energy and nutrient intake was analyzed with NutriSurvey software version 2016 (EBISpro, Stuttgart, Germany).

### 2.7. Questionnaires

The patients completed questionnaires at baseline and at the final post-treatment dental visit. These validated and reliable questionnaires were collected in order to evaluate the changes between before and after the full-mouth rehabilitation. A standardized questionnaire from the bulimia outpatient clinic was collected from each patient with a focus on their disease and dental changes (e.g., hypersensitivity, dental pain). The body image questionnaire (FKB-20) for patients with body image disorders was used to determine negative body appearance and physical vitality [31]. The WHO quality-of-life questionnaire (WHOQOL-BREF) was used to assess the quality of life concerning physical health, psychological aspects, social relationships, and environment [32].

### 2.8. Statistical Analysis

Given the exploratory nature of this retrospective case series and the small sample size, the statistical analyses were primarily descriptive. Data are described as the mean and standard deviation (SD), or median and range, and percentage. The normality of the differences was assessed prior to analysis. As the differences were normally distributed, a paired *t*-test was used to compare measurements between the baseline visit (T1) and the post-treatment visit, defined as the last full-mouth rehabilitation visit (T2). Given the retrospective design and the small, specialized patient cohort treated with this novel dental intervention, no a priori sample size calculation was performed.

All tests were two-tailed, with statistical significance being accepted at *p* < 0.05. Data analysis was carried out using the software product SPSS version 26.0 (SPSS Inc, IBM Company, Chicago, IL, USA).

### 2.9. Ethical Considerations

This study was approved by the local Institutional Review Board of the Medical University of Vienna (EK No. 2363/2020). The requirement for written informed consent was waived, as the study consisted exclusively of a retrospective analysis of pre-existing clinical data. Nevertheless, all patients were fully informed about the novel digital workflow and were given adequate time to decide whether to undergo the planned full-mouth rehabilitation. Written informed consent for treatment, as well as for the use of pseudonymized clinical data and images for research purposes, is routinely obtained from all patients at the clinic, including those included in this study.

## 3. Results

### 3.1. Study Population

In total, nine adults (11% male) with dental erosion were part of this cohort. The patients were, on average, 36 years old (mdn = 34; 29–54), with an average body height of 170 cm (mdn = 169; 165–177) and an average body mass index (BMI) of 19 kg/m^2^ (mdn = 18; 15–29). Seven patients (78%) were underweight, one (11%) was normal weight, and one patient (11%) was overweight according to the WHO criteria [33]. The severe erosion was caused in seven (78%) patients by bulimia nervosa, in one (11%) patient by reflux, and in one (11%) patient by the excessive consumption of soft drinks over many years. The bulimia nervosa patients suffered from this eating disorder for 16 years on average (mdn = 15; 6–30) and had undergone psychotherapy for 5 years (mdn = 3; 0–18). These patients indicated vomiting on average four times a day (mdn = 2; 1–10). The bulimia nervosa patients were affected by the following comorbidities: two (29%) patients had depression, two (29%) had anorexia nervosa, one (14%) had osteopenia, one (14%) had bruxism, and one (11%) patient had amenorrhea. Both patients with erosion caused by reflux and soft drinks had bruxism.

### 3.2. Digital Workflow via a Four-Step Technique

In all patients, the average time from the baseline visit (T1) to the final FMR visit (T2) was eight months (mdn = 7; range 3–15). The increase in the vertical dimension of occlusion was carefully evaluated to ensure functional adaptation and patient comfort. The vertical dimension of occlusion was increased by +5 mm in 33% (3/9), by +7 mm in 45% (4/9), and by +10 mm in 22% (2/9) of the patients. At the baseline visit, the oral diagnostics of the patients showed typical consequences of erosive tooth wear like unstable occlusal contacts, inadequate centric and eccentric contacts, erosive loss of tooth substance, disharmonic tooth length–width relation, and inappropriate red–white aesthetics.

With the support of the novel digital workflow, oral changes like stable occlusion, restoration of adequate centric and eccentric contacts, biomimetics of an adequate dental anatomy, harmonic tooth length–width relation, and optimization of the red–white aesthetics could be achieved in these patients.

**Figure 11:**
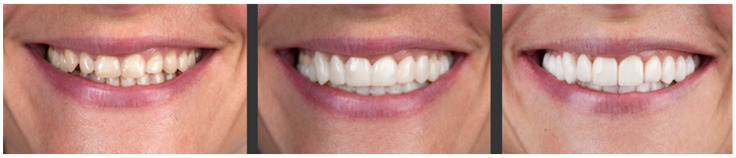
Severe tooth wear, removable sample dentures, and non-prep restorations.

The following illustration presents the severe tooth wear at the beginning of the dental treatment, the removable sample dentures (prototypes), and the non-prep restorations with single crowns giving a new vertical dimension of occlusion (Figure 11, from left to right).

**Figure 12:**
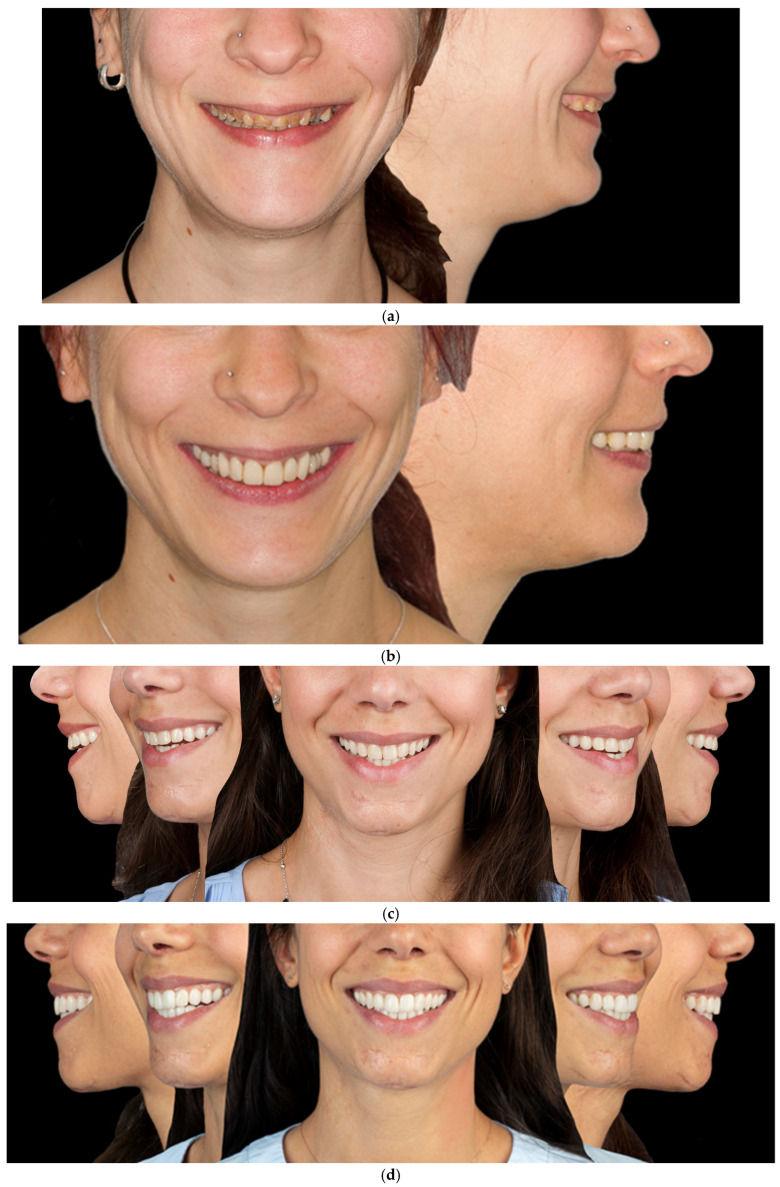

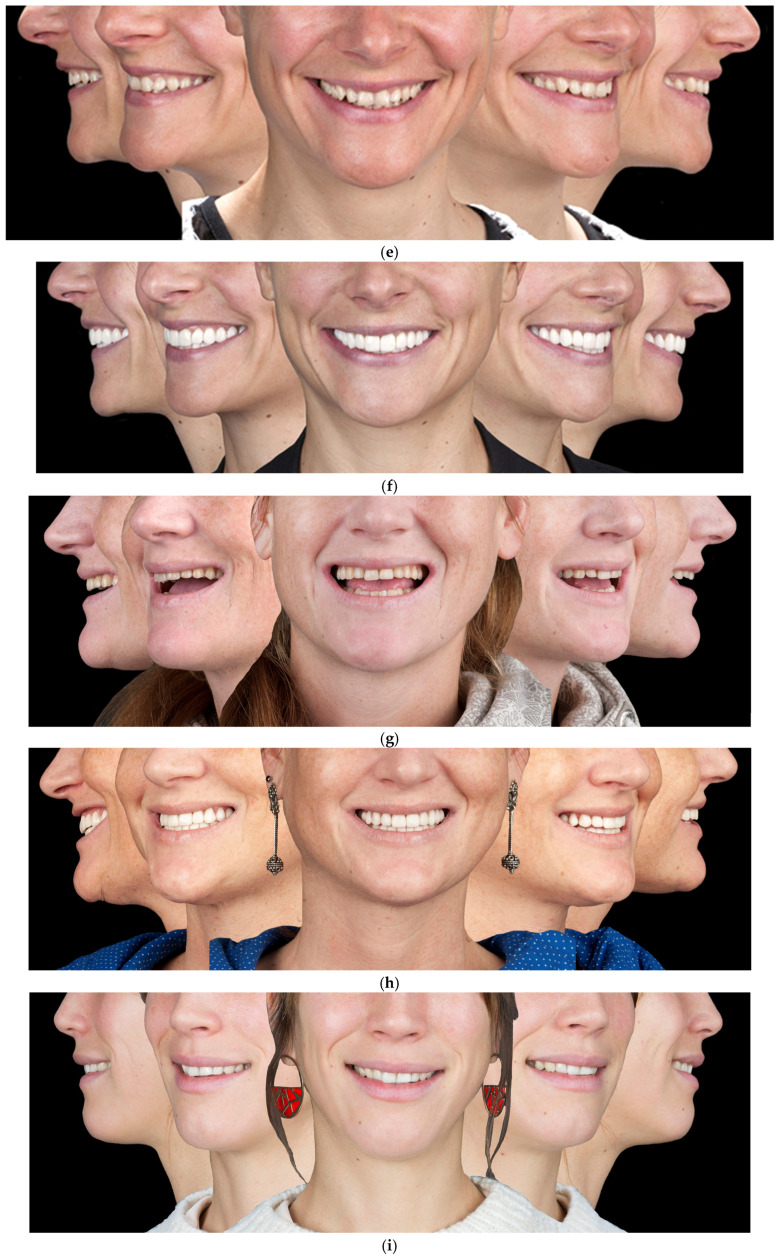

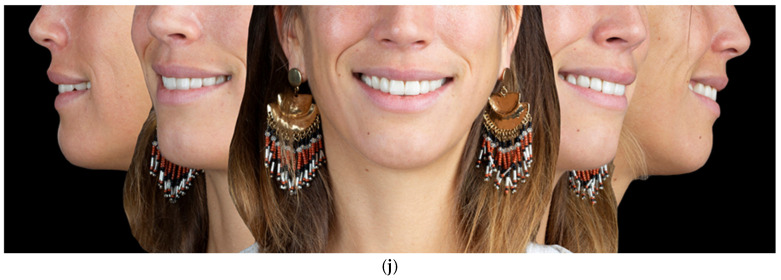
(**a**) Patient 1 before full-mouth rehabilitation (FMR). (**b**) Patient 1 after full-mouth rehabilitation (FMR). (**c**) Patient 2 before full-mouth rehabilitation (FMR). (**d**) Patient 2 after full-mouth rehabilitation (FMR). (**e**) Patient 3 before full-mouth rehabilitation (FMR). (**f**) Patient 3 after full-mouth rehabilitation (FMR). (**g**) Patient 4 before full-mouth rehabilitation (FMR). (**h**) Patient 4 after full-mouth rehabilitation (FMR). (**i**) Patient 5 before full-mouth rehabilitation (FMR). (**j**) Patient 5 after full-mouth rehabilitation (FMR).

**Figure 13:**
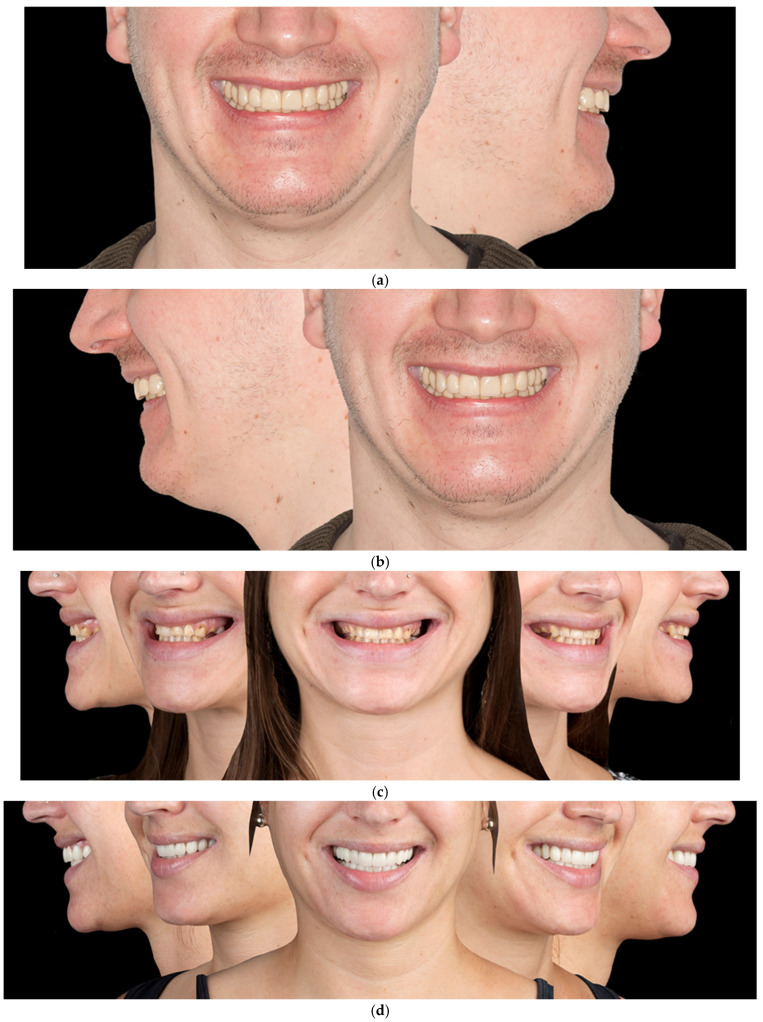

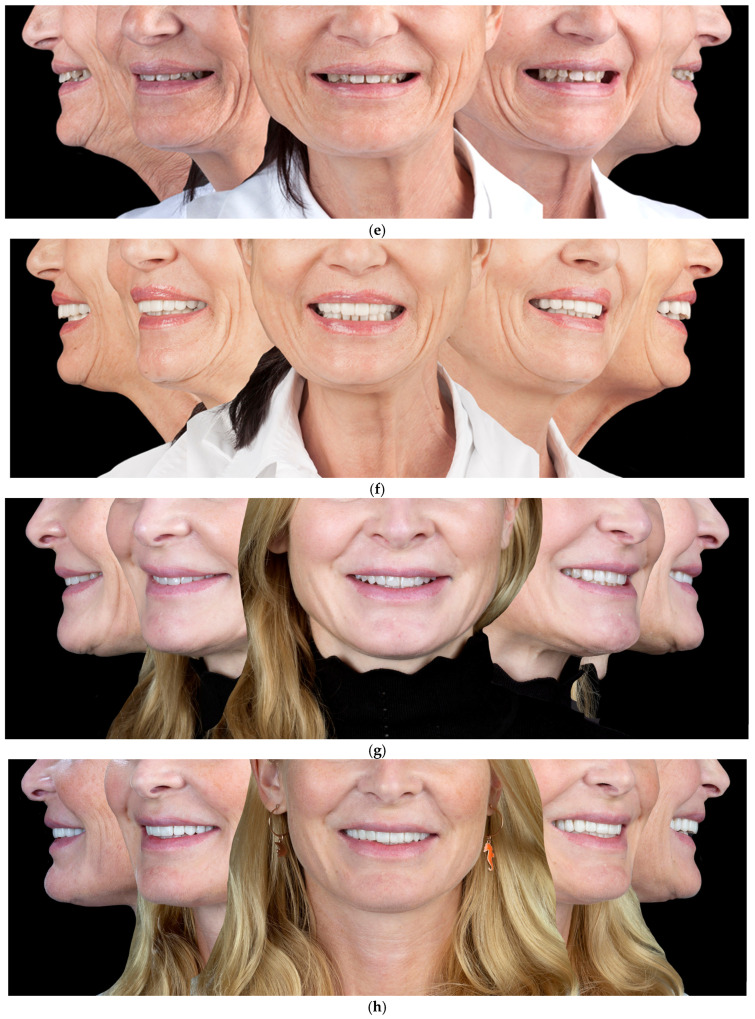
(**a**) Patient 6 before full-mouth rehabilitation (FMR). (**b**) Patient 6 after full-mouth rehabilitation (FMR). (**c**) Patient 7 before full-mouth rehabilitation (FMR). (**d**) Patient 7 after full-mouth rehabilitation (FMR). (**e**) Patient 8 before full-mouth rehabilitation (FMR). (**f**) Patient 8 after full-mouth rehabilitation (FMR). (**g**) Patient 9 before full-mouth rehabilitation (FMR). (**h**) Patient 9 after full-mouth rehabilitation (FMR).

The following figures (Figure 12 and Figure 13) illustrate the individual patients and their development and changes before and after the implementation of the structured full-mouth rehabilitation, carried out using the novel digital workflow presented here for patients with severe dental erosion.

### 3.3. Nutrition

The patients went from underweight to normal weight after their FMR. In this study, dietary intake and eating behavior changed between baseline and finalization of the full-mouth rehabilitation. The daily macronutrients and energy intake significantly decreased (*p* < 0.02), as did the numbers of binge-eating and following vomiting episodes per day (*p* < 0.05). Moreover, monthly expenses for food and drinks significantly decreased (*p* < 0.03), although out-of-home food consumption (restaurants and canteen) with social contact (colleagues and friends/family) increased (*p* > 0.05) (Table 2).

### 3.4. Dental, Body-Image and Quality of Life Questionnaires

In all, 89% (8/9) of the patients completed the questionnaires and nutritional interview at baseline and after the dental therapy. Out of three smokers, one patient stopped smoking after the dental treatment. At baseline, 63% (5/8) had toothache when chewing and 75% (6/8) suffered from tooth sensitivity; both complaints disappeared completely after the dental therapy. Diet, negative body evaluation, vital body dynamics, and quality of life improved, especially in the bulimia nervosa patients. Self-confidence was increased in 75% (6/8) of the patients, who now like to show their teeth, especially with an open smile, and found their face to be more attractive.

## 4. Discussion

This retrospective case series showcased a structured approach to full-mouth rehabilitation using an innovative digital workflow for full-mouth rehabilitation in patients with severe dental erosion, focusing on restoration of an individualized vertical dimension of occlusion and functional and aesthetic maxillomandibular stability, alongside evaluations of patient-reported outcomes including dental symptoms, nutritional behavior, facial and dental self-perception, and quality of life. Implementation of this four-step digital workflow enabled individualized VDO adjustment, stable occlusion, restoration of functional and aesthetic dental parameters, and optimization of red–white aesthetics. Patients with severe dental erosion, due to conditions such as bulimia nervosa, reflux, or prolonged excessive consumption of soft drinks, experienced reduced dental hypersensitivity and pain, as well as improvements in their nutrition, body perception, and overall quality of life after treatment.

### 4.1. Standardized Full-Mouth Rehabilitation

Conventional full-mouth rehabilitation restorative concepts are realized via the preparation of all teeth for restoration with crowns. This has the disadvantages of possible tooth devitalization and root canal treatment, with worse prognosis and lower success rates for the treated teeth. Another disadvantage is the lack of predictability regarding the final restoration’s aesthetics and function, which means higher stress for the patient and for the dentist. The advantages of the innovative therapeutic approach are its structured and stepwise treatment planning, “gradual” non-invasive introduction to the changes, and non-invasive/minimally invasive restorations that preserve the natural tooth structure.

Structured treatment planning represents a key factor in the success of full-mouth rehabilitation. While predictability is an important concept in full-mouth rehabilitation, the present retrospective case series design does not allow definitive conclusions regarding predictability of clinical outcomes. The findings should therefore be interpreted as preliminary and hypothesis generating. Simultaneous restoration of the entire dentition is inherently complex and carries the risk of functional complications, aesthetic dissatisfaction, or temporomandibular disorders. The proposed digital workflow addresses these challenges by enabling objective evaluation at several treatment stages. Accurate digital replication of the intraoral situation provides a reliable basis for treatment planning, while individualized determination of the VDO integrates cephalometric and soft-tissue analyses. In the present study, the increase in VDO (+5 mm, +7 mm, or +10 mm) was individualized for each patient based on a comprehensive clinical assessment, including baseline VDO, estimated loss of tooth structure, functional considerations, and patient-reported comfort. Occlusal splints were used exclusively chairside to evaluate different VDO increments and were not prescribed for home use. The final VDO was selected based on the patient’s subjective perception of comfort in combination with clinical functional assessment.

A non-invasive trial phase using removable sample dentures allows functional and aesthetic verification and facilitates patient feedback prior to definitive treatment. The validated prototype is subsequently transferred to final restorations using non-prep or minimal-prep crowns, ensuring highly precise and reproducible outcomes.

Dental erosion typically affects multiple teeth rather than isolated sites, emphasizing the importance of treatment strategies that minimize biological cost in accordance with the ALARA principle [34]. Preservation of tooth structure is an important treatment objective. Therefore, non-preparation approaches may represent a suitable option for patients with dental erosion, as not all teeth necessarily require further reduction through crown preparation.

Increasing the vertical dimension of occlusion can provide several clinical benefits, including improved occlusal relationships, increased restorative space, and enhanced aesthetic outcomes through restoration of lower facial height and soft tissue support [35]. Current evidence suggests that the mandibular rest position exists within a physiological “comfort zone,” and moderate increases in VDO (<5 mm) are generally well tolerated by the stomatognathic system when carefully indicated and controlled. This adaptability has been demonstrated in both dentate and prosthetically rehabilitated patients, with neuromuscular adaptation representing the primary compensatory mechanism [35]. However, increasing VDO is not without risks. Short-term adverse effects may include muscular discomfort, altered masticatory function, phonetic difficulties, and perceived aesthetic changes, particularly during the initial adaptation phase. A recent systematic review reported that removable devices used for VDO evaluation may contribute to chewing impairment and unclear speech, although the available evidence remains limited regarding their impact on long-term clinical outcomes [23]. Furthermore, the risk of temporomandibular symptoms or functional instability may increase if the magnitude of change exceeds the patient’s adaptive capacity or if modifications are introduced too rapidly. Although recent scoping reviews have not established a definitive causal relationship between altered VDO and temporomandibular disorders, ongoing controversy and variability in clinical interpretation persist [36].

Given these considerations, contemporary clinical practice emphasizes a controlled and patient-centered approach. A reversible trial phase using interim appliances or diagnostic restorations is recommended to evaluate individual tolerance prior to definitive treatment. This strategy enables clinicians to balance expected benefits against potential risks while accounting for patient-specific functional adaptation. This individualized and evidence-based rationale underpins the chairside determination of VDO and the progressive adaptation protocol employed in the present study.

Given the retrospective nature of the study, accurate quantification of prior tooth substance loss was not possible. Consequently, standardized increments in the vertical dimension of occlusion were applied to ensure consistency and comparability across cases. The functional and aesthetic effects of the VDO modification were confirmed through chairside clinical assessment.

Full-mouth rehabilitation is essential in patients with dental erosion at a progressed stage [19,20,21,37]. However, FMR leads to significant changes in function and aesthetics.

Hence, certain predictions of the outcome are made to improve the prognosis for success of the FMR for both the patient and the dentist. The simultaneous restoration of 28 teeth is a complex treatment and could lead to potential failures. It is already a complete failure when the perception of the restoration’s final outcome differs between dentist and patient (e.g., the color of the teeth), even if it has been performed medically correctly. Failure occurs not only when aesthetic questions are unsolved but also when the restoration shows dental malfunctions, pain occurs that did not exist before, or the patient experiences temporomandibular joint problems. In order to prevent negative consequences on both sides in terms of health, time, and financial losses, it is advantageous to utilize this structured FMR approach with the principle “begin with the end in mind.” A factor in its structured evaluation process is the possibility to test-drive the function and aesthetics, which helps to identify the expectations on both sides in advance and supports the patient in becoming accustomed to the new oral changes with this dental appliance.

In two out of the nine cases presented here, a complete fixed restoration was not recommended. In these patients, severe erosive lesions were found in addition to gaps between the teeth as a result of tooth loss. Both of these patients were suffering from an active phase of bulimia nervosa. It is not recommended to place dental implants during an active bulimic phase. Therefore, the irregular dentition was corrected using a combination of removable dentures and dental crowns. The four-step technique was also performed on these patients. The consistency of the treatment approach was supported with the removable restoration as the aesthetic and function of the digital prototype set-up were reused for the final outcome. The final outcome was realized through CAD/CAM support with a combination of non-prep crowns, minimal-prep crowns, and regular-prep crowns with removable partial dentures.

In one patient, the removable partial dentures were supported through the CAD of Ultaire AKP for Ceramill^®^ (Amann Girrbach, Germany) and were milled out of Solvay Dental 360^®^ by Ceramill^®^ Motion 2 (Amann Girrbach, Germany). The second patient received a CAD-supported conventionally manufactured print-cast out of chrome–cobalt–molybdenum alloy remanium^®^ GM 800 (Dentaurum, Germany).

For complex cases involving active bulimia or interdental gaps due to tooth loss, full fixed restorations were avoided. Instead, a combination of removable partial dentures and crowns was used, while maintaining predictability through the digital test-drive and CAD/CAM-supported fabrication. This workflow ensured reproducible functional and aesthetic outcomes, highlighting the clinical feasibility and patient-centered benefits of a digitally guided, minimally invasive approach in challenging full-mouth rehabilitation cases.

### 4.2. Material

In comparison to other ceramic materials, ENAMIC^®^ (VITA Zahnfabrik, Bad Säckingen, Germany), as a hybrid material, has the following beneficial factors: The benefit of CAM fabrication is the time saved in production by efficient polishing without any firing process. The hybrid material accepts sharper edges of the tooth surface because of its high degree of elasticity, so crowns can be produced on non-prepped/minimally prepped teeth. The crown surface can be modified through an additive workflow, which is beneficial in case of potential modifications to the occlusion surface (i.e., occlusal contacts) or to repair any possible chipping of the material. Non-invasive/minimally invasive restorations are possible since the hybrid ceramic enables reduced wall thicknesses; thus, the natural tooth structure can be preserved by the reconstruction. After the full-mouth rehabilitation with hybrid ceramic material, a night guard to protect parafunctions was given to each patient. The use of these night guards was highly recommended every night to prevent chipping and fracturing of the hybrid ceramic restorations.

### 4.3. Patients with Bulimia Nervosa

Patients with bulimia nervosa reported previous participation in psychotherapy, with some continuing therapy at the time of the study. Participation in psychotherapy was not a requirement for dental treatment. No changes in ongoing psychotherapy were reported during the FMR. Additionally, no dietary counseling or nutritional education was provided before or during the dental intervention.

Overall, the nutritional behavior of the bulimia patients changed significantly. At baseline, patients with bulimia nervosa were characterized by underweight status and reduced BMI despite a markedly elevated energy intake exceeding 9000 kcal per day. This was due to the high number of binge-eating and vomiting attacks. After the dental intervention, all bulimia nervosa patients stopped their regular binge-eating and vomiting episodes, with one exception—this patient still suffers from body scheme disorder. The patients showed an age-appropriate BMI. This is a great success in the therapy of patients with eating disorders and shows that this novel four-step technique has more than only positive dental effects. Multiple reasons behind this outcome should be mentioned: The sample denture prototypes were a vomiting threshold for the bulimia nervosa patients. The patients reported that the sample dentures had to be removed and cleaned after vomiting every episode. This additional effort was burdensome and time-consuming. Thus, the prototypes turned out to be a preventive tool for vomiting reduction or even to stop vomiting. The prototypes also played an important role as motivation not to ruin the future dental restoration, again by vomiting. In addition, the mouth, as the location that was previously negatively affected by excessive food consumption, vomiting, a destroyed smile, and unhealthy teeth, was turned into a well-being zone during the dental treatment. In the well-being zone, reasonable dietary intake without vomiting episodes was represented. The decrease in or even discontinuation of binge-eating vomiting episodes led to a reduction in food intake, further leading to a decrease in excessive food shopping and, therefore, to a reduction in financial expenses. Moreover, it caused an exchange from high-quantity to high-quality food. The gradual improvement of the oral condition and the final outcome were further reasons why the patients refrained from this eating disorder. The change to an attractive smile is associated with better self-perception, increased self-esteem, and increased social contact [38,39,40,41], which was observed in this present study. A shift from loneliness and isolation to cultivation of social contacts in association with eating was noticed. Dining in restaurants was possible again and could also be enjoyed with friends, family, and colleagues.

### 4.4. Limitations

This study has several limitations inherent to its retrospective case-series design. First, the absence of a control group precludes any conclusions regarding causality. Second, patient-reported outcomes are subjective and prone to bias, including recall and perception effects. Third, the small sample size is a notable limitation of this study, which may weaken the robustness of statistical inferences and restrict the generalizability of the findings. Finally, the lack of systematic follow-up constitutes an additional limitation; future prospective studies should incorporate comprehensive follow-up assessments to better evaluate long-term outcomes. Nevertheless, these limitations reflect the clinical reality of managing patients with severe dental erosion associated with bulimia nervosa, who often present late and may be reluctant to disclose the underlying eating disorder due to stigma. Consequently, well-documented cohorts remain limited in the literature. Furthermore, full-mouth rehabilitation cases involving the controlled vertical dimension of occlusion increase require complex and time-intensive treatments, which further restricts the number of cases available for systematic clinical evaluation. Despite these limitations, this study provides valuable preliminary data and insights that can inform future research, providing a basis for future controlled studies.

## 5. Conclusions

The presented digital workflow represents a structured and clinically applicable approach for full-mouth rehabilitation in patients with severe dental erosion, particularly associated with bulimia nervosa. It enables stepwise evaluation of functional and aesthetic parameters and supports patient-centered treatment planning and adaptation.

However, due to the retrospective design and limited sample size, no definitive conclusions regarding predictability can be drawn. Future prospective studies with larger cohorts are required to validate the long-term clinical outcomes and patient-reported benefits of digitally guided full-mouth rehabilitation in this specific patient population.

## Figures and Tables

**Table 1:** Treatment plan for complex cases with dental erosion.

Complex Case	Complex Demands	Solution: Four-Step Technique
Patients with a chronic psychological and/or physiological disorder	Reduction in number of overstrained appointments	Step 1: One single computer-aided impression
Discrepancy of the vertical dimension of occlusion	Determination of a new vertical dimension of occlusion	Step 2: Cephalometric analysis and soft tissue prediction
Changed facial appearance caused by a new vertical dimension of occlusion, which can lead to difficulties in perception (particularly for patients with body scheme disorders like bulimia nervosa)	Structured evaluation and test-driving of the restoration	Step 3: Removable sample dentures—prototypes
Erosive loss of dental hard tissues	Less invasive/non-invasive approach (to maintain natural tooth structure)	Step 4: Non-prep/minimal-prep crowns

**Table 2:** Changes in nutrition of severe dental erosion patients at baseline and after FMR.

	Baseline	After FMR	*p*-Value
BMI (kg/m^2^)	18.2 ± 2.2	19.1 ± 0.8	0.301
**Daily macronutrient intake**			
Protein (g)	336 ± 151	54.4 ± 15.2	**0.015**
Fat (g)	473 ± 181	50.9 ± 35.8	**0.005**
Carbohydrates (g)	840 ± 346	137 ± 40	**0.012**
Energy (kcal)	9002 ± 3554	1498 ± 265	**0.009**
**Eating behavior**			
Binge eating per day	4 ± 3	0.2 ± 0.4	**0.043**
Vomiting per day	4 ± 3	0.2 ± 0.4	**0.043**
Expenses for food/drinks per month (€)	460 ± 114	258 ± 199	**0.021**
Out-of-home eating per week	3 ± 4	6 ± 3	0.074
Eating with colleagues per week	1 ± 1	2 ± 1	0.367
Eating with friends/family per week	0.5 ± 0.7	2 ± 1	0.059

Results are given as mean ± SD. Comparisons were made using paired-sample *t*-test. Bold font indicates statistical significance.

## Data Availability

Data that support the findings of this study are available upon reasonable request.

## Abbreviations

The following abbreviations are used in this manuscript:

BN	Bulimia nervosa
TMJ	Temporomandibular joint
VDO	Vertical dimension of occlusion
FMR	Full-mouth rehabilitation
MCA	Maximum credible accident
CAI	Computer-aided impression
CAD	Computer-aided design
CAM	Computer-aided manufacturing
PMMA	Polymethyl methacrylate
SD	Standard deviation
BMI	Body mass index
